# Classifying epileptic phase-amplitude coupling in SEEG using complex-valued convolutional neural network

**DOI:** 10.3389/fphys.2022.1085530

**Published:** 2023-01-05

**Authors:** Chunsheng Li, Shiyue Liu, Zeyu Wang, Guanqian Yuan

**Affiliations:** ^1^ Department of Biomedical Engineering, School of Electrical Engineering, Shenyang University of Technology, Shenyang, China; ^2^ Department of Electrical Engineering and Information Systems, University of Pannonia, Veszprem, Hungary; ^3^ Department of Neurosurgery, General Hospital of Northern Theater Command, Shenyang, China

**Keywords:** epilepsy, SEEG, complex-valued phase-amplitude coupling, complex-valued convolutional neural network, epileptogenic zone

## Abstract

EEG phase-amplitude coupling (PAC), the amplitude of high-frequency oscillations modulated by the phase of low-frequency oscillations (LFOs), is a useful biomarker to localize epileptogenic tissue. It is commonly represented in a comodulogram of coupling strength but without coupled phase information. The phase-amplitude coupling is also found in the normal brain, and it is difficult to discriminate pathological phase-amplitude couplings from normal ones. This study proposes a novel approach based on complex-valued phase-amplitude coupling (CV-PAC) for classifying epileptic phase-amplitude coupling. The CV-PAC combines both the coupling strengths and the coupled phases of low-frequency oscillations. The complex-valued convolutional neural network (CV-CNN) is then used to classify epileptic CV-PAC. Stereo-electroencephalography (SEEG) recordings from nine intractable epilepsy patients were analyzed. The leave-one-out cross-validation is performed, and the area-under-curve (AUC) value is used as the indicator of the performance of different measures. Our result shows that the area-under-curve value is .92 for classifying epileptic CV-PAC using CV-CNN. The area-under-curve value decreases to .89, .80, and .88 while using traditional convolutional neural networks, support vector machine, and random forest, respectively. The phases of delta (1–4 Hz) and alpha (8–10 Hz) bands are different between epileptic and normal CV-PAC. The phase information of CV-PAC is important for improving classification performance. The proposed approach of CV-PAC/CV-CNN promises to identify more accurate epileptic brain activities for potential surgical intervention.

## 1 Introduction

Epilepsy is the most common chronic disease in neurology. About 70% of patients with epilepsy can be cured by taking antiepileptic drugs, and other patients may develop drug-resistance epilepsy (DRE) (Kwan and Brodie, 2000). The epileptogenic zone (EZ) is the brain region responsible for seizure generation (Rosenow and Luders., 2001). Some patients with DRE can be treated by surgical intervention on EZ (Engel, 2019). Scalp electroencephalography (EEG) is one of these techniques which is fundamental for defining the EZ, frequently as a precursor to invasive recordings. Intracranial EEG (iEEG) signal provides anatomically precise information about the selective engagement of neuronal populations at the millimeter scale and about the temporal dynamics of their engagement at the millisecond scale (Parvizi and Kastner, 2018). Stereo-electroencephalography (SEEG) is one kind of iEEG and is widely used to study the spatiotemporal oscillatory dynamics of brain networks engaged in epileptogenic processes (Bartolomei et al., 2017). Some EEG features provide promise biomarkers for EZ, such as phase-amplitude coupling (PAC) (Weiss et al., 2015; Amiri et al., 2016; Jiang et al., 2019; Liu et al., 2021), and high-frequency oscillations (Worrell and Gotman, 2011). Currently, there is still a gap between those studies and their applications in the clinical setting. Machine learning can be used to close the gap in supporting clinical applications.

EEG PAC, where the amplitude of high-frequency oscillations (HFOs) is modulated by the phase of low-frequency oscillations (LFOs), is a useful biomarker to identify the activities of epileptogenic tissue (Guirgis et al., 2015). Cross-frequency push-pull dynamics contributed to the secondary generalization of focal seizures and potentially reflected impaired excitation-inhibition interactions of the epileptic network (Jiang et al., 2019). PAC feature of ictal EEG is used to determine the region of interest in epilepsy (Guirgis et al., 2015). The coupling phase was suggested as an interictal marker of the seizure-onset zone (SOZ) (Amiri et al., 2016). The PAC in the inter- and pre-seizure periods was weak and paroxysmal, and strong PAC channels were confined more to the SOZ and resection region (Ma et al., 2021). The theta—gamma PAC within the electrodes in the seizure region increased during the ictal period (Liu et al., 2021). In Parkinson’s disease, the HFO (100–300 Hz) was found modulated by beta (13–30 Hz), and beta and gamma amplitudes were further modulated by their low-frequency components (Jin et al., 2022). Some studies have shown that cross-frequency coupling (CFC) plays a functional role in physiological functions, such as memory, and task performing (Lisman and Idiart, 1995; Canolty and Knight, 2010). It is difficult to apply those coupling patterns for identifying pathological brain tissues. To identify pathological PAC is critical for further applications in the clinical setting (von Ellenrieder et al., 2016). A multistage classifier based on the random forest was applied to classify CFC features and it successfully predicted seizures (Jacobs et al., 2018). Different kinds of algorithms have been applied in investigating epileptic EEG, such as principal component analysis (PCA) (Villar et al., 2017), Wavelet analysis (Wang et al., 2018), support vector machines (SVM), fuzzy logic systems (Jiang et al., 2016), and connectivity (Qin et al., 2020).

Convolutional neural networks (CNN) becomes more popular in neuroscience research after its success in some other fields, such as image recognition (Krizhevsky et al., 2012) and EEG analysis (Pan et al., 2022). CNN achieves automatic extraction of local features through its key component convolutional kernel and obtains high-level abstract features after a series of hierarchical processing. It may also avoid the problems of manual optimizing of traditional signal processing algorithms. In our preliminary study, a three-layer CNN was trained to identify pathological PAC in SEEG recordings (Wang and Li, 2020). The result showed that the area-under-curve (AUC) value reached .88 for classifying pathological PACs from normal ones (Wang and Li, 2020). However, the representation and operation of CNN in real values limit their applications in the field of complex-valued datasets. Complex-valued CNN (CV-CNN) has been developed and applied to various fields (Hirose, 2013; Tygert et al., 2016). Some studies have demonstrated that CV-CNN outperforms real-valued CNN after making full use of phase information in complex-valued data, such as magnetic resonance imaging (MRI) (Cole et al., 2021), steady-state visually evoked potentials (SSVEP) (Ravi et al., 2020).

In this study, we propose a novel approach for identifying pathological PAC in SEEG from patients with epilepsy. We first provide a method for generating complex-valued PAC (CV-PAC) with both the coupling strength and the coupled phase of LFO. The CV-CNN is then trained to discriminate the pathological PACs from normal ones. SEEG recordings from nine intractable epilepsy patients were further analyzed to validate our proposed approach.

## 2 Materials and methods

### 2.1 Data and subject description

SEEG data of 23 seizures from nine patients were used in this study. All patients had undergone surgery and achieved seizure-free outcomes (General hospital of northern theater command). The regions of surgical resection were used as the epileptogenic zone in this study. Informed consent was obtained from each patient, and the ethics committee of the hospital approved the study. The clinical information of each patient is outlined in Table 1. A neurologist marked the SEEG onset and termination of all seizures. To eliminate the reference effect, we transform the SEEG recordings into a bipolar montage. Channels with obvious artifacts are removed based on visual inspection. EEG is a non-linear and non-stationary signal. It could be treated as a stable state within a short duration. A 10 s window was suggested for computing PAC (Guirgis et al., 2015; Shi et al., 2019). We used a 10 s sliding window on seizure with a step size of 2 s. The duration of each seizure is listed in Table 1. There are 38,751 CV-PACs generated. Each CV-PAC is represented as a complex-valued image. The CV-PAC is labeled as a pathological pattern if the corresponding SEEG channel resides in the surgical resection. Otherwise, the CV-PAC is labeled as normal. There are 10,289 CV-PACs marked as pathological, and the other 28,462 CV-PACs are marked as normal.

**TABLE 1:** Clinical information of patients studied in this work.

Patients	Age/Sex	Duration (years)	Seizure (s)	MRI Findings	Pathology	Surgery
P1	16M	10	66, 61, 48	Left hippocampal abnormality	FCD/HS	Left: T
P2	37M	17	74, 76, 77	Normal	FCD	Left: T
P3	36M	23	75, 84	Normal	Gliosis	Right: T
P4	31M	30	42, 64, 120	Multiple region abnormality	Gliosis	Right: T
P5	25F	17	65, 97, 67	Normal	HS	Right: T
P6	38M	32	71	Left occipital abnormality	—	Left: O*
P7	11M	7	44, 54, 55	Left parietal and right occipital abnormality	Gliosis/FCD	Right: O
P8	54M	32	59, 60, 82	Right temporal abnormality	FCD	Right: FT
P9	22M	3	76, 80	both hippocampal abnormality	HS	Left: T

F, frontal; T, temporal; O, occipital; *, radio-frequency thermo-coagulation; —, unknown; FCD, focal cortical dysplasia; HS, hippocampal sclerosis.

### 2.2 Complex-valued phase-amplitude coupling

The CV-PAC is generated based on the PAC measure (Guirgis et al., 2015). In this study, the low-frequency range is chosen as 1–10 Hz, and the high-frequency range is chosen as 30–160 Hz. Both low- and high-frequency ranges are further divided into 10 intervals equally in log space. We denote the selected low- and high-frequency signals as $x_{f_{P}}\left(t\right)$ and $x_{f_{A}}\left(t\right)$ (i.e., $A_{f_{A}}\left(t\right)$ ) and the instantaneous phase of $x_{f_{P}}\left(t\right)$ (i.e., $\Phi_{f_{P}}\left(t\right)$ ) is extracted by using continuous wavelet transformation (CWT) in MATLAB (MathWorks, Natick, USA). The phases $\Phi_{f_{P}}\left(t\right)$ are binned and the mean of $A_{f_{A}}$ over each phase bin is calculated, which denotes as $<A_{f_{A}}{>}_{\Phi_{f_{P}}}\left(j\right)$ . The mean amplitude is then normalized by the sum of all mean amplitudes in each phase bin *j*, as follows

$$P\left(j\right)=\frac{<A_{f_{A}}{>}_{\Phi_{f_{P}}}\left(j\right)}{\overset{N}{\underset{k=1}{\sum }}<A_{f_{A}}{>}_{\Phi_{f_{P}}}\left(k\right)},$$ (1)

where *N* = 18, and *j* is chosen from 1 to 18. The Kullback-Leibler (KL) distance between amplitude distribution *p* and uniform distribution *U* (*U*(*j*) = 1/*N* for all bins *j*) is measured by following equation (Tort et al., 2010):

$$D_{KL}\left(P,U\right)=\sum_{j=1}^{N}P\left(j\right)*\log\left[\frac{P\left(j\right)}{U\left(j\right)}\right].$$ (2)

The strength of CV-PAC at low- and high-frequency pair is calculated as follows

$$S_{\mathrm{PAC}}=\frac{D_{KL}\left(P,U\right)}{\log\left(N\right)}.$$ (3)

The phase bin at the peak of *P*(*j*) is extracted as the coupling phase of the corresponding high and low frequency pair

$$\psi_{\mathrm{PAC}}=\underset{j}{\arg\max}P\left(j\right)*\frac{\pi }{9}.$$ (4)

The *S*_*PAC*_ is also called modulation index (MI) (Tort et al., 2010). The strength *S*_*PAC*_ and phase *ψ*_*PAC*_ are then used as the module and phase angle of complex-valued vector

$$C_{\mathrm{PAC}}=S_{\mathrm{PAC}}*\cos\left(\psi_{\mathrm{PAC}}\right)+i*S_{\mathrm{PAC}}*\sin\left(\psi_{\mathrm{PAC}}\right)$$ (5)

where *i* is the imaginary number. *C*_*PAC*_ forms one pixel in CV-PAC image.

The surrogate-tested CV-PAC (ST-CV-PAC) is also generated. The phases in each low frequency are shuffled 100 times. If the coupling strength of *C*_*PAC*_ in ST-CV-PAC is lower than the maximum 5% of the corresponding shuffled values, the *C*_*PAC*_ will be set to 0. The CV-PAC and CV-PAC-SA in 10 × 10 resolution are used in this study since our preliminary study shows that it is a good balance between performance and computational load (Wang and Li, 2020).

**FIGURE 1:**
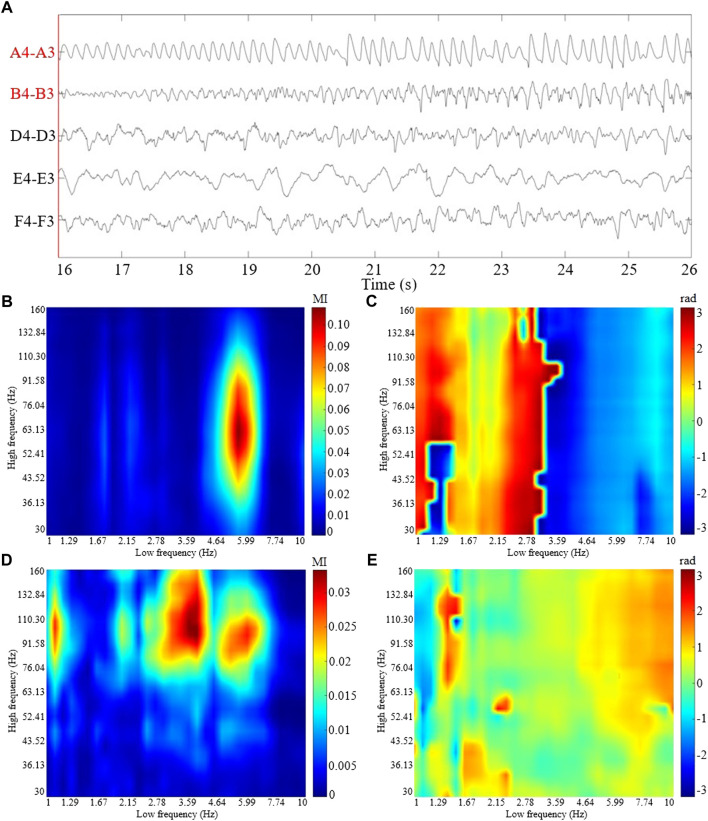
The ictal SEEG segment and the CV-PAC patterns from patient P1. **(A)** 10 s SEEG segment with selected channels 16 s after seizure onset. The labels of the SEEG channel in surgical resection are marked in red color. **(B)** The coupling strength of CV-PAC of channel A4-A3 in surgical resection. Red and blue colors indicate strong and weak coupling strengths, respectively. The value is between 0 and 1. **(C)** The coupled phase of CV-PAC of channel A4-A3 corresponding to **(B)**. The range of phase is between −*π* and *π* rad. **(D)** The coupling strength of CV-PAC of channel D4-D3 in the normal brain region. Red and blue colors indicate strong and weak coupling strengths, respectively. **(E)** The coupled phase of CV-PAC of channel D4-D3 corresponding to **(D)**. The range of phase is between −*π* and *π* rad.

A 10 s SEEG segment during seizure is shown in Figure 1A. The coupling strengths and coupled phases of CV-PACs for pathological and normal activities are shown in Figures 1B–E, respectively. To compare the performance of CV-CNN with traditional CNN, SVM, and random forest, the real part of CV-PAC and the imaginary part of CV-PAC are used as two-layer images when using CNN to classify PAC patterns. The PAC with only coupling strength is also used to train traditional CNN for comparison.

### 2.3 Complex-valued convolutional neural network

**FIGURE 2:**
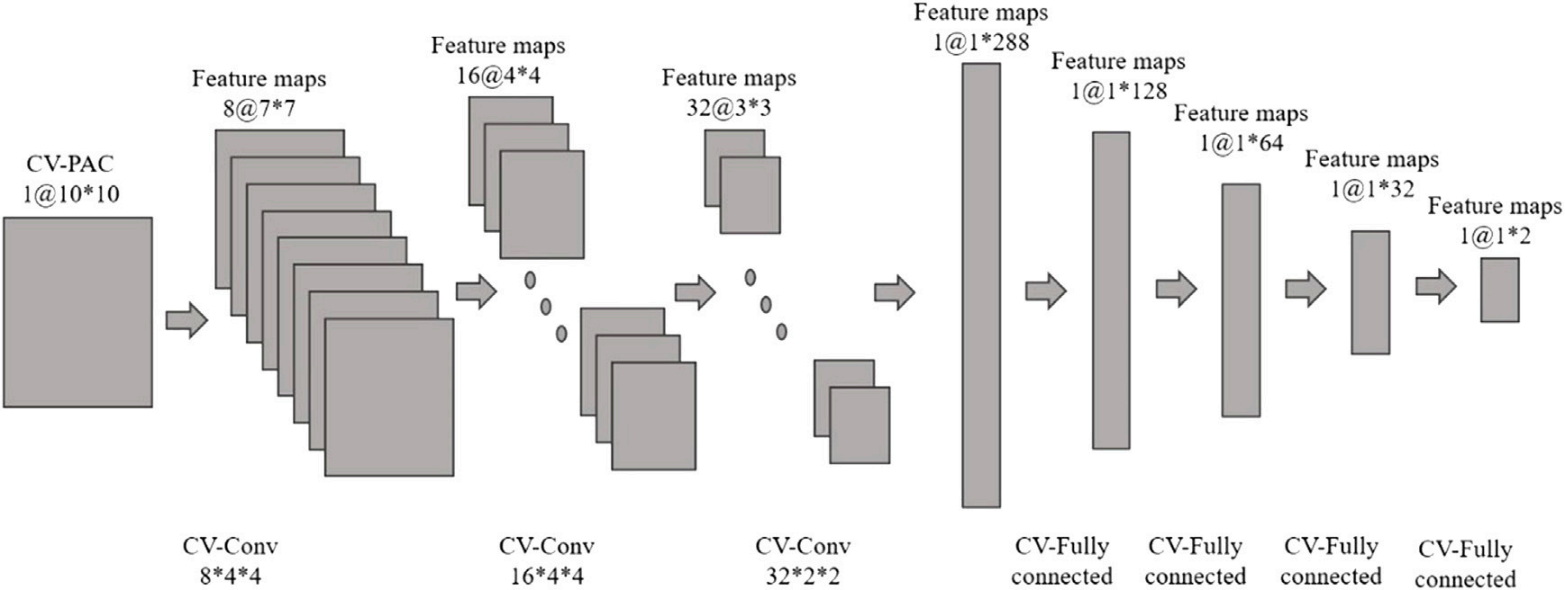
Complex-valued convolutional neural network (CV-CNN) with complex-valued phase-amplitude coupling (CV-PAC). There are three complex-valued convolutional layers and four complex-valued fully connected layers.

In this study, we use CV-CNN to classify CV-PAC patterns. The structure of CV-CNN is shown in Figure 2. The activation function of CV-CNN is implemented using the rectified linear function (ReLU) in our study. The ReLU is applied on the real and the imaginary feature maps separately. In each complex-valued convolutional layer, the weights of the convolution kernel are complex values, and complex multiplication between weights and feature maps is implemented. To speed up the training convergence of the model and reduce the impact of the variation of the input, we define a complex batch normalization (BN) layer. The maximum number of iterations epoch set for training is 800, the batch size is 128, and the learning rate is .0025. In addition, the learning rate decays by a factor of .5 when the epoch is an integer multiple of 250. A dropout with a value of .2 is used in the first layer of the fully connected layer, and the weights of all the complex convolution layers are regularized using L2 with *λ* = .004. The cross-entropy loss function is used to quantify the loss, and the stochastic gradient descent (SGD) algorithm is chosen as the optimization function. The training and testing of dataset are implemented using the PyTorch package.

## 3 Result

### 3.1 Identifying channel with pathological CV-PAC

**FIGURE 3:**
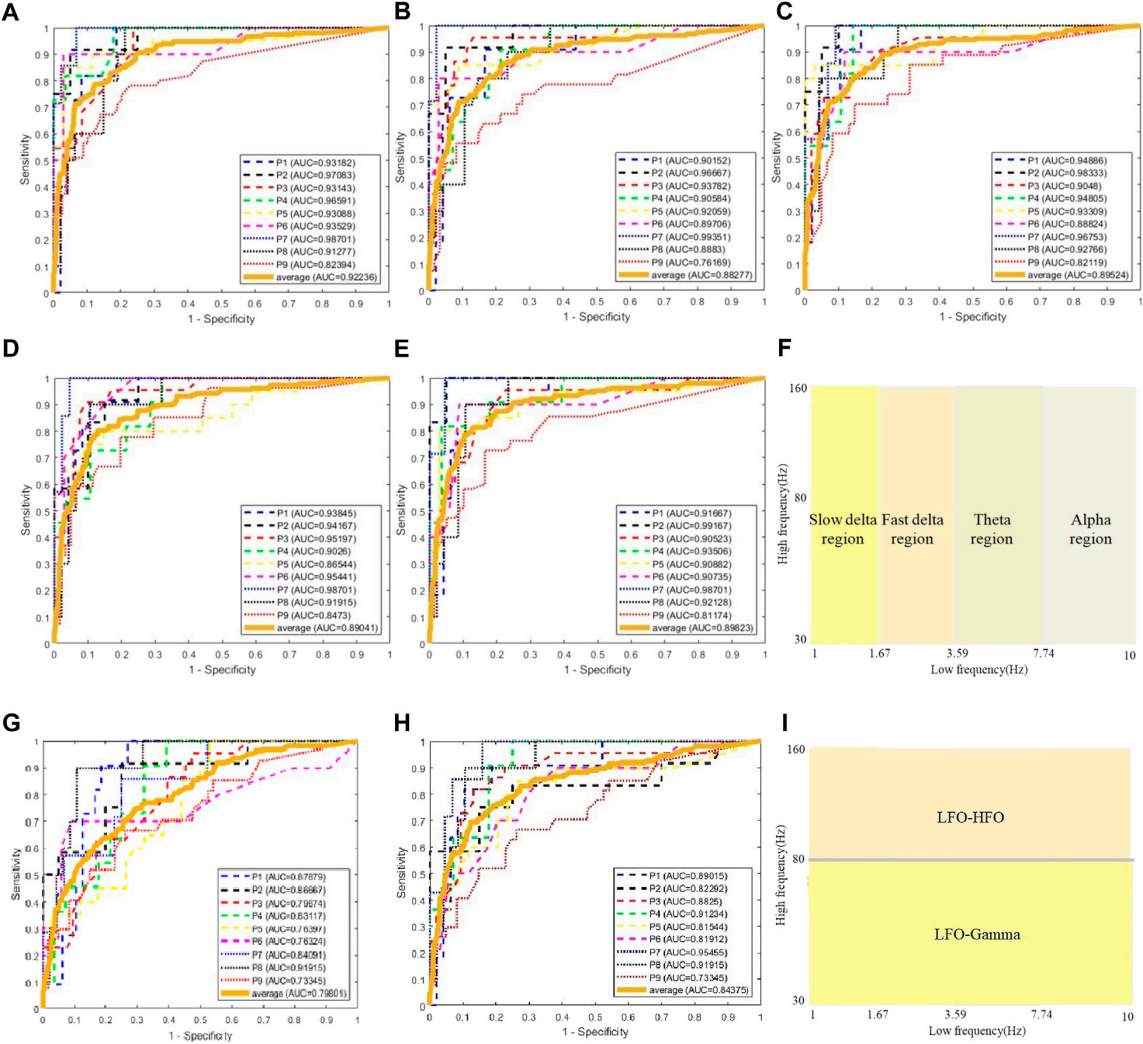
ROC curves of CV-CNN on CV-PAC dataset. **(A)** CV-PAC with full low-frequency band. **(B)** CV-PAC without slow delta. **(C)** CV-PAC without fast delta. **(D)** CV-PAC without theta **(E)** CV-PAC without alpha. **(F)** PAC regions are divided into four low-frequency bands. **(G)** CV-PAC with LFO-gamma. **(H)** CV-PAC with LFO-HFO. **(I)** PAC is divided into LFO-gamma and LFO-HFO regions.

We use the AUC value to evaluate the performance of classification. To classify one channel as pathological or normal is based on all CV-PAC generated from the channel. In the receiver operating characteristic (ROC) curve, if the percentage of pathological CV-PAC from one channel is higher than the optimal threshold, the channel will be classified as pathological. The leave-one-out cross-validation is performed. CV-PACs of eight patients are used as the training set to train the CNN model, and the remaining patient is used as a test. A total of nine rounds of training and verification are performed. By comparing with the ground truth of each CV-PAC, the ROC curve is obtained, as shown in Figure 3A. The AUC values of all patients are listed in the first column of Table 2. The average AUC value is .92 when applying CV-CNN on the CV-PAC dataset. The sensitivity and specificity are .82 and .83, respectively.

**TABLE 2:** AUC values using CV-CNN on CV-PAC.

Patients	CV-PAC	CV-PAC without slow delta	CV-PAC without fast delta	CV-PAC without theta	CV-PAC without alpha	CV-PAC with LFO-gamma	CV-PAC with LFO-HFO
P1	.93182	.90125	**.94886**	.93845	.91667	.87879	.89015
P2	.97083	.96667	.98333	.94167	**.99167**	.86667	.82292
P3	.93143	.93782	.90480	**.95197**	.90523	.79674	.88250
P4	**.96591**	.90584	.94805	.90260	.93506	.83117	.91234
P5	.93088	.92059	**.93309**	.86544	.90882	.76397	.81544
P6	.93529	.89706	.88824	**.95441**	.90735	.76324	.81912
P7	.98701	**.99351**	.96753	.98701	.98701	.84091	.95455
P8	.91277	.88830	**.92766**	.91915	.92128	.91915	.91915
P9	.82394	.76169	.82119	**.84730**	.81174	.73345	.73345
Average	**.92236**	.88277	.89524	.89041	.89823	.79801	.84375

Optimal values at each row are shown in bold.

To further investigate the effects of each low-frequency band, the PAC is divided into four regions, as shown in Figure 3F. The low-frequency bands include slow delta (1—2 Hz), fast delta (2—4 Hz), theta (4—8 Hz), and alpha (8—10 Hz). Each time we replace one of the CV-PAC regions with random values with the same mean and variance. The ROC curves are drawn in Figures 3B–E. The AUC values decrease to .882, .895, .890, and .898 for the slow delta, delta, theta, and alpha region replaced, respectively. The high-frequency band is then divided into gamma (30–80 Hz) and HFO (80—160 Hz), as shown in Figure 3I. The result shows that the AUC values decrease to .80 and .84 for using LFO-gamma and LFO-HFO regions (Figures 3G, H; Table 2), respectively. It is also interesting to notice that the test on original CV-PACs achieves the best performance on patient P4 only.

**FIGURE 4:**
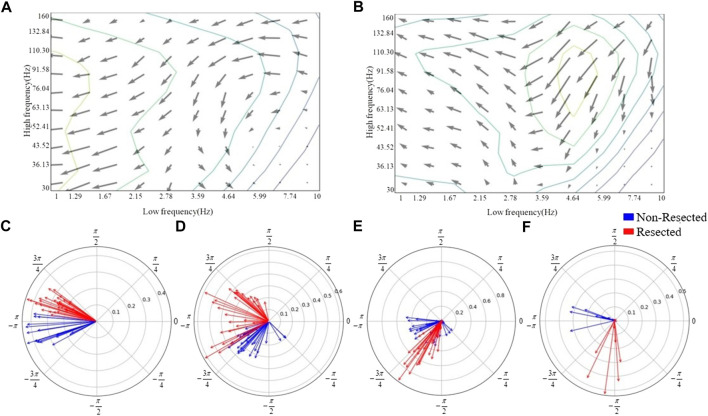
Phase distribution of low-frequency bands. **(A)** The quiver map of averaged strength and phase of CV-PACs in non-resected regions. Note that the length and angle of each gray arrow represent the coupling strength and coupled phase at each high- and low-frequency pair, respectively. **(B)** The quiver map of averaged strength and phase of CV-PACs in resected regions. The length and angle of each arrow represent the coupling strength and coupled phase, respectively. **(C)** The coupling with phases in slow-delta (1–2 Hz). The blue and red arrows indicate the coupling in the non-resected and resected regions, respectively. **(D)** The coupling with phases in delta (2–4 Hz). **(E)** The coupling with phases in theta (4–8 Hz). **(F)** The coupling with phases in alpha (8–10 Hz).

The CV-PACs from non-resected regions and resected regions are averaged separately. The coupling strengths and coupled phases of pathological and normal CV-PAC are plotted in Figures 4A, B, respectively. The two patterns are different. The coupled phases in low-frequency bands are plotted in Figures 4C–F. The coupled phases in non-resected and resected regions are separable for slow delta, fast delta, and alpha bands. The phases in the non-resected and resected regions are overlapped for theta band, but the coupling strengths in the resected regions are stronger than the ones in non-resected regions. These features contribute to the capability of identifying pathological CV-PAC in the resected regions.

### 3.2 Comparison with CNN, SVM, and RF

**FIGURE 5:**
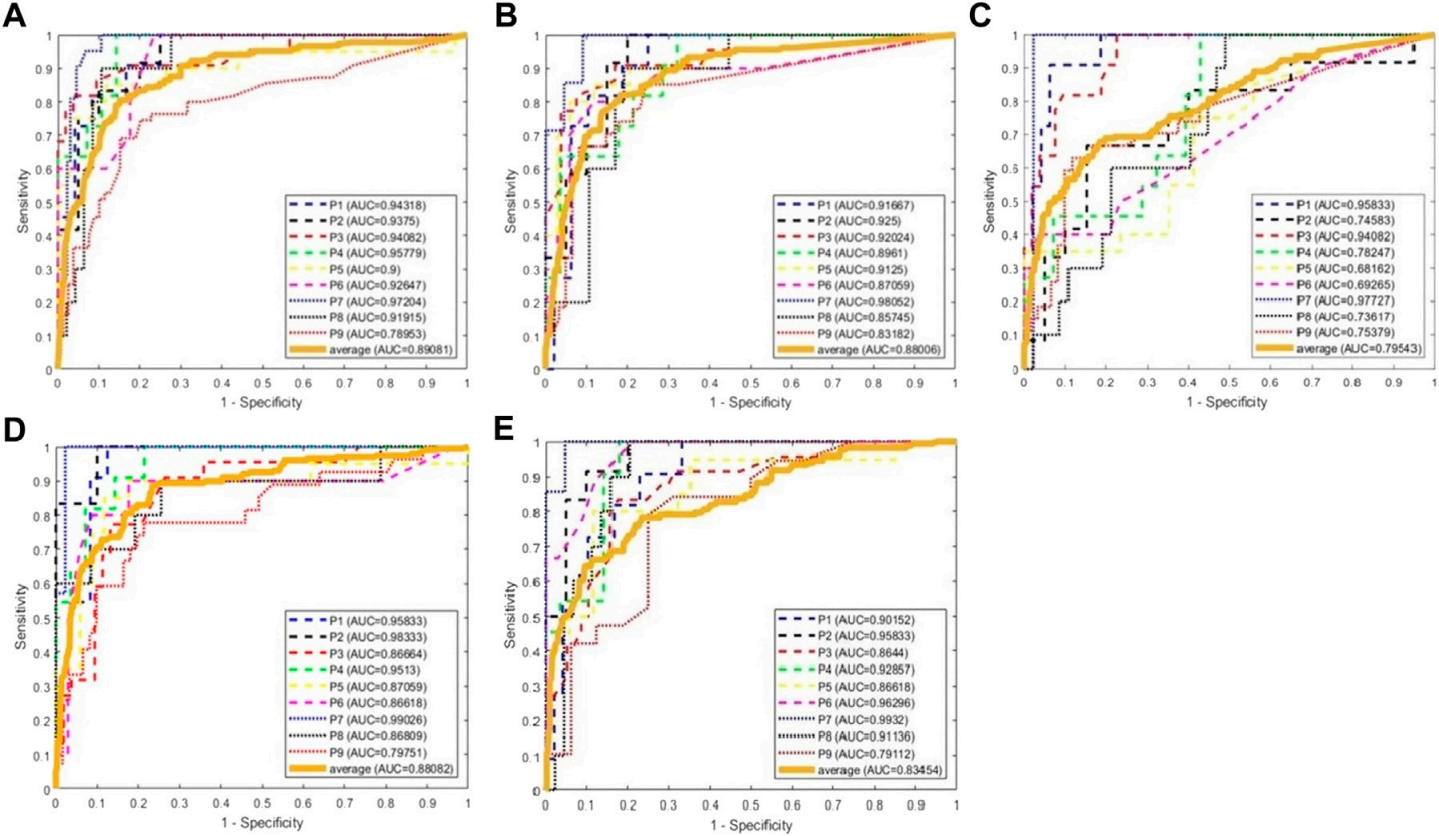
ROC curves of classification using CNN, SVM, random forest, and CV-CNN. **(A)** ROC curves using CNN. **(B)** ROC curves using SVM. **(C)** ROC curves using random forest. **(D)** ROC curves using CNN and PAC. **(E)** ROC curves using CV-CNN and ST-CV-PAC.

We use traditional CNN, SVM, and random forest (RF) to train the model. The leave-one-out cross-validation is also performed. The real part and imaginary part of CV-PAC are extracted to form images with two layers. The obtained three-dimensional vectors are used as the features of the RF classifier. The number of trees searched is from 50 to 300. Since the performance does not improve after 100 trees, the number of trees is set to 100 in the remaining tests. The features used for SVM are the same as the RF. The SEEG of patient P1 is first trained and tested using the SVM method. A grid search over the parameters *C* (2^2^, 2^6^, …, 2^20^) and *γ*(2^–10^, 2^–8^, …, 2^10^) is performed to find optimal values. The parameter *C* is set to 2^18^, and the parameter *γ* is set to 2^2^. Those values of parameter *C* and *γ* are used for further analysis. The ROC curves are plotted in Figures 5A–C, and the AUC values are .890, .795, and .880 for traditional CNN, SVM, and RF, respectively. The performances of the above methods are lower than CV-CNN, as shown in Table 3. The PACs (without the coupled phases) are used to train the traditional CNN. The ROC curves are plotted in Figure 5D, and the AUC value is .88 (Table 3). We use the ST-CV-PAC to train the CV-CNN model with leave-one-out cross validation. The ROC curves are plotted in Figure 5E, and the averaged AUC value is .83 (Table 3).

**TABLE 3:** Comparisons of AUC values by using CNN, SVM, RF, CNN(PAC), and CV-CNN(ST).

Patient	CNN	SVM	RF	CNN(PAC)	CV-CNN(ST)
P1	.94318	**.95833**	.91667	**.95833**	.90152
P2	.93750	.74583	.92500	**.98333**	.95833
P3	**.94082**	**.94082**	.92024	.86664	.86440
P4	**.95779**	.78247	.89610	.9513	.92857
P5	.90000	.68162	**.91250**	.87059	.86618
P6	.92647	.69265	.87059	.86618	**.96296**
P7	.97204	.97727	.98052	.99026	**.99320**
P8	**.91915**	.73617	.85745	.86809	.91136
P9	.78953	.75379	**.83182**	.79751	.79112
Average	**.89081**	.79543	.88006	.88082	.83454

Optimal values at each row are shown in bold.

## 4 Discussion

The PAC is commonly used to localize the epileptic tissue (Weiss et al., 2015; Liu et al., 2021), but the coupled phase of LFO in PAC is seldom used due to the complexity of the pattern. The proposed CV-PAC contains both the coupling strengths and the coupled phases. Our result shows that the CV-PAC/CV-CNN approach outperforms PAC/CNN approach. The AUC values of the two approaches are .92 and .88, respectively (Tables 2, 3). When the real part and imaginary part of CV-PAC are extracted as two-layer input feature maps for traditional CNN, the AUC value decreases to .88. The performance will not improve if we just feed the CNN with real-value features. It implies that the coupling strengths and the coupled phases are correlated, and the complex-value operation in CV-CNN extracts the correlated information for identifying pathological PAC.

The coupled phases of LFOs are important in localizing the epileptogenic tissues (Amiri et al., 2016; Li et al., 2016). In our study, the delta band is divided into slow delta and fast delta (Amiri et al., 2016). Our result showed that the coupled phases of both slow delta and fast delta are different in pathological and normal PACs, as shown in Figures 4C, D. If the coupling in slow delta region was replaced by random values, the performance of classification dropped the most, as shown in Table 2. Our result also showed that the coupling in alpha region also contributes to the improvement of performance (Table 2; Figure 4F). The coupled phases in theta band are overlapped in pathological and normal PAC (Figure 4E). The coupling strengths of pathological PACs in delta band are stronger than the coupling strengths of normal PACs (Figures 4A, B). We infer that it is the main reason why the traditional PAC or MI can help us discriminate pathological brain tissues.

Some studies focused on the PAC in either LFO-gamma (30–80 Hz) or LFO-HFO ( >= 80) (Amiri et al., 2016; Liu et al., 2021). Our result shows that the AUC values scored only .80 and .84 when using CV-PAC of LFO-gamma (30–80 Hz) and LFO-HFO (80–160 Hz), respectively. Our result suggests that the CV-PAC of LFO-HFO (80–160 Hz) is more important than the CV-PAC of LFO-gamma (30–80 Hz) in classifying pathological patterns.

In our study, the PAC in the form of two-layer feature maps was used to train the SVM and RF. The performance of RF is comparable to PAC/CNN approach (Table 3). The AUC value of CV-PAC/CV-CNN approach is higher than all other methods, as listed in Table 3, which emphasizes the importance of the correlation between the coupling strength and coupled phase. The coupled phase is important, and it is more meaningful when combined with coupling strength. Since there are difficulties in analyzing the phase patterns of PAC, our proposed approach provides a tool for the classification of pathological PAC and normal PAC by introducing a complex-value image classification measure. Surrogate testing can be used to remove the spurious coupling in EEG signals (Shi et al., 2019; Li et al., 2021). In our study, the averaged AUC value using ST-CV-PAC is lower than the value using CV-PAC. Here, we adopt the image recognition measure by using CNN and CV-CNN. The ST-CV-PAC may become more complex and discontinuous due to removing some non-significant values. We think that is the main reason why the performance on ST-CV-PAC is lower than CV-PAC.

There are some limitations in this study. The ictal dataset is analyzed, and it still needs more study to extend it to inter-and pre-seizure data. Since the subdural is another widely used measure for recording brain electrical activity, it is necessary to include those types of data. In our study, most patients had temporal lobe epilepsy, and the location of SEEG implemented varied from patient to patient. It should be cautious to apply our method to all candidates for epilepsy surgery. Some studies have shown that PAC can be used to identify channels in SOZ, which is often much smaller than the area surgically removed. Comparing our results with traditional SOZ classification results still needs to be further explored. The non-linear decomposition methods for generating PAC may potentially improve the classification performance, which will be addressed in our future work.

## 5 Conclusion

The PAC pattern is a useful biomarker for identifying SEEG channels with pathological brain activities, and it is critical for presurgical evaluation of DRE patients. The proposed CV-PAC represents richer pathological patterns than PAC, which can be further analyzed by using image recognition measure. The CV-CNN achieves better performance than traditional machine learning measures in classification of pathological and normal PAC patterns. This study provides a new approach for localizing epileptogenic brain tissues.

## Data Availability

The raw data supporting the conclusion of this article will be made available by the authors, without undue reservation.
